# Evidence of Anxiety, Depression and Learning Impairments following Prenatal Hypertension

**DOI:** 10.3390/bs12020053

**Published:** 2022-02-18

**Authors:** Kedra Wallace, Teylor Bowles, Ashley Griffin, Reanna Robinson, Lucia Solis, Teryn Railey, James P. Shaffery, Sarah Araji, Shauna-Kay Spencer

**Affiliations:** 1 Department of Pharmacology & Toxicology, University of Mississippi Medical Center, Jackson, MS 39216, USA; sspencer2@umc.edu; 2 Department of Obstetrics & Gynecology, University of Mississippi Medical Center, Jackson, MS 39216, USA; teylorbowles@yahoo.com (T.B.); reannalrobinson2016@gmail.com (R.R.); lsolis@umc.edu (L.S.); terynrailey1@gmail.com (T.R.); saraji@umc.edu (S.A.); 3 Department of Neurobiology & Anatomical Sciences, University of Mississippi Medical Center, Jackson, MS 39216, USA; 4 Program in Neuroscience, University of Mississippi Medical Center, Jackson, MS 39216, USA; agriffin4@umc.edu; 5 Department of Psychiatry, University of Mississippi Medical Center, Jackson, MS 39216, USA; jpshaffery@umc.edu

**Keywords:** anti-angiogenic, memory, HELLP syndrome, placental ischemia, pregnancy, post-partum, preeclampsia

## Abstract

Background: Hypertensive disorders of pregnancy, such as Preeclampsia (PreE) and HELLP (hemolysis, elevated liver enzyme, low platelet) syndrome, affects approximately 5–10% of pregnancies and increases the risk of women developing disorders, such as anxiety or depression, in the postpartum period. Using preclinical rodent models, we set out to determine whether rats with a history of PreE or HELLP had evidence of anxiety, depression or cognitive impairment and whether immune suppression during pregnancy prevented these changes in mood and/or cognition. Methods: Timed-pregnant rats were infused with sFlt-1 and/or sEng to induce PreE or HELLP beginning on gestational day 12. After delivery, a battery of validated behavioral assays was used to assess post-partum depression, anxiety and learning. Results: There was no negative effect on maternal pup interaction due to PreE or HELLP; however, hypertensive dams spent more time immobile in the forced swim test (*p* < 0.0001). Hypertensive dams also spent less time in the open area of the open field (*p* = 0.001). There were no significant changes in recognition memory (*p* = 0.08); however, spatial learning was impaired in hypertensive dams (*p* = 0.003). Immobility time in the forced swim test was positively correlated with increased circulating S100B (*p* = 0.04), while increased time spent in the outer zones of the open field was negatively correlated with BDNF levels (*p* < 0.0001). Conclusion: The results from this study suggest that hypertensive pregnancy disorders are associated with depression, anxiety and learning impairments in the post-partum period.

## 1. Introduction

Hypertensive disorder of pregnancy (HDP) affects approximately 5–10% of all pregnancies [1,2]. Women with a history of HDPs are at an increased risk of developing mood disorders, such as anxiety and depression [3,4,5]. Women with advanced HDPs, such as preeclampsia (PreE; new onset of high blood pressure after 20 weeks gestation) or HELLP (hemolysis, elevated liver enzyme, low platelet; considered to be a variant of PreE with more severe organ dysfunction and often life threatening) syndrome, are even more susceptible to these mood disorders and have been reported by some to have disturbances in cognitive function [6,7,8]. There have been several theories as to why women affected by HDPs are more susceptible to these changes, ranging from cerebral vascular dysfunction during pregnancy to long-term complications due to hypertension [9,10]; however, to date, none have been conclusive. What is more striking is that despite the similar etiology between PreE and HELLP, the few reports on maternal mood and cognition differ between the two conditions.

Few studies have examined the relationship between HDP and mood disorders, such as depression, anxiety and cognition. Of those that have, it has been reported that 7–44% of women develop post-partum depression, and 26–32% of women develop post-partum anxiety following a HDP [5,11,12,13,14]. The changes in cognition have been even more varied depending on the length of time that had passed and the type of memory that was evaluated. However, the overall consensus is that HDP does have a negative influence on learning and memory in the post-partum period [15,16].

PreE and HELLP were both found to cause a dysregulation of the immune system and anti-angiogenic factors [17,18,19]. In particular, anti-angiogenic factors resulting from placental ischemia have been proposed to contribute to the development of inflammation and hypertension in these disorders [20,21,22,23]. Several clinical and experimental studies have provided evidence that the hypertension and systemic inflammation present in PreE and HELLP syndrome are also accompanied by blood–brain barrier disruption [24,25,26,27]. Furthermore, attenuation of hypertension and systemic inflammation leads to a reduction in blood–brain barrier damage, suggesting that neuroinflammation and perhaps any subsequent behavioral disruptions are also prevented [26,27]. We have previously published data indicating that a blockade of T-cell activation during pregnancy prevents anxiety-like behavior in HELLP rats when evaluated prior to post-partum week 4 [28]. In this former study, HELLP rats buried significantly more marbles in the marble-burying task and spent significantly more time in the closed arms of the elevated plus maze; both behaviors were reversed to levels comparable to normal pregnant rats when T-cell activation was blocked during pregnancy in HELLP rats. However, we did not assess post-partum depression or cognitive dysfunction in relation to neuroinflammation, nor did we evaluate a preclinical model of PreE. In the present study, our primary objective was to determine whether rats with a history of HDPs have evidence of depression, anxiety and impaired learning. In addition, we also sought to determine whether a blockade of T-cell activation during pregnancy prevents any negative changes in mood and/or learning and whether these changes are due to improvements in neuroinflammation during the post-partum period.

## 2. Materials and Methods

All studies were performed using Sprague Dawley rats from Envigo Laboratories (Indianapolis, IN; 230–250 g on arrival at gestational day 11) housed in a temperature-controlled room with a 12:12 reverse light:dark cycle. All experimental procedures in this study were in accordance with the National Institute of Health guidelines for use and care of animals and were approved by the Institutional Animal Care and Use Committee at the University of Mississippi Medical Center under protocol 1414A.

### 2.1. Preclinical Models of HDP

**Figure 1:**
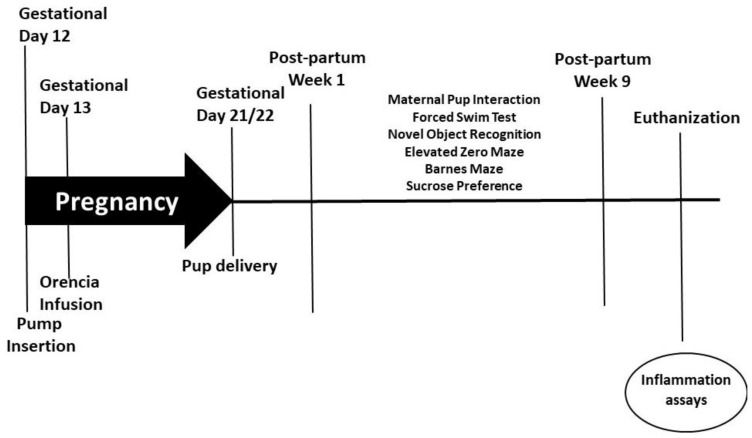
Study design and timeline of behavioral assays and experiments.

On gestational day 12, timed-pregnant rats (230–250 g) had mini-osmotic pumps (model 2002, Alzet Scientific Corporation; Cupertino, CA, USA) inserted intra-peritoneally while under anesthesia. To induce PreE, the pumps were loaded with soluble fms-like tyrosine kinase 1 (sFlt-1; 4.7 µg/kg, R&D Systems, Minneapolis, MN, USA), and HELLP pumps were loaded with sFlt-1 and soluble endoglin (sEng; 4.7 and 7 µg/kg, respectively, R&D Systems), as previously described [17,18,19]. Normal pregnant (NP) rats underwent abdominal sham surgeries at this same time period. To prevent T-cell activation during pregnancy, Orencia (Abatacept, 2 mg/kg) was infused into a subset of rats via the jugular vein on gestational day 13, as previously described [27,28,29]. Rats gave birth between gestational day 21 and 22, at which time the pups were weighed and litters culled to 4 each, with median weighted pups being kept. Behavioral testing was conducted for 9 weeks starting from post-partum week (PPW) 1 after which the animals were euthanized. The study timeline is outlined in Figure 1. The following numbers of animals were used in the current study: NP (n = 13), PreE (n = 13), HELLP (n = 12), NP + Orencia (n = 12), PreE + Orencia (n = 11), HELLP + Orencia (n = 13).

### 2.2. Behavioral Test

All behavioral analyses were performed primarily by TB, RR and LS who were blind to experimental conditions. Each cohort consisted of a mixed group of rats, so that all groups were equally exposed to any environmental fluctuations. Unless noted otherwise, all animals had an hour to acclimate to the testing environment prior to testing. Finally, all testing was performed under low-light conditions during the active phase for the animals. Behavioral testing was recorded and tracked using Noldus Observer unless otherwise notated. A battery of tests was used for each behavioral domain. Depression/Anhedonia, Anxiety and Cognition were assessed.

#### 2.2.1. Post-Partum Depression and Anhedonia

Maternal Pup Interaction. Beginning on post-partum day 3 until post-partum day 7, dams were removed from cages for 30 min and then reintroduced to their litter of 4 pups, who were previously culled. Following reintroduction, the interaction between dams and pups was recorded for a period of 10 min. All testing occurred twice a day during each cycle (light and dark) over the duration of 5 days (total of 100 min of observation per dam). All testing occurred in the housing room, and testing variation between testing times was kept to a minimum. To ensure blinding, all dams were renumbered prior (KW) to testing (TB, LS), and the code was not broken until after the video tapes were scored (SKS and AG, r^2^ = 0.98). The following behaviors were scored: maternal behavior (included licking and/or sniffing pups) or nursing of pups (arch-back, blanket nursing and side); self-maintenance behavior consisted of self-grooming or self-feeding; and neglecting behavior was considered to be tunneling through the bedding or no contact [30]. Scorers recorded the incidence of each behavior over the 10 min testing period. Maternal behavior was analyzed as the frequency (%) of observations in which the dam engaged in the behavior over the testing period. At the end of maternal pup interaction testing, all pups were removed from dams. The dams were pair housed for the duration of the study 48–72 h following pup removal.

Forced Swim Test. The forced swim test is a classical test used to evaluate depressive-like behavior in rodents [31]. The test is based on the measurement of immobility time (i.e., time spent floating) in a tank filled with water. The apparatus consists of glass cylinders containing 20–22 cm of fresh tap water at 23 °C. On the day of testing, the rats were placed in the tank for a 5 min period. All videos were analyzed for the swimming and immobility time.

Sucrose Preference. Before the test, the animals were deprived of food and water for 20 h. During the test each animal was presented simultaneously with two pre-measured bottles: one containing 2% sucrose solution and the other tap water. The total amount of water per solution was measured after 48 h, and the percent preference for sucrose consumption was calculated. Sucrose preference (%) was determined as follows = sucrose solution consumption/(sucrose solution consumption + water consumption) × 100 [28]. Plexiglass dividers with holes were placed in the cages, which gave animals the ability to interact with their cage mate while still only drinking from their individual water source(s).

#### 2.2.2. Post-Partum Anxiety

Open-field test. Locomotor and exploratory behavior were assessed in the open-field arena. The rats were placed in the center of the open field under bright lights and allowed to freely explore for 20 min. The amount of time rats spent exploring the center (open area) of the arena vs. remaining on the perimeter was measured. At the end of the trial, the animals were returned to their home cages.

Zero maze. Rats were placed in the open arm of the zero-maze platform and allowed to explore for a total of 5 min. Rodents were observed for the amount of time spent in the open and closed arms of the maze. At the end of the trial, the animals were returned to their home cages.

#### 2.2.3. Post-Partum Learning and Memory

Novel Object Recognition. Novel object recognition was used to assess recognition memory [32]. Rats were placed in a chamber box with two identical objects for 5 min. The amount of time spent exploring the objects was recorded. After an inter-trial interval, the rats were placed in the chamber again, and this time they had 5 min to explore 1 familiar object and 1 novel object. The amount of time spent exploring both objects was recorded. The discrimination index, or time spent with the novel object, was calculated by

[(novel − familiar)/(novel + familiar)] × 100.

Barnes Maze. Spatial learning and memory was assessed over 5 consecutive days in the Barnes maze [33]. At the beginning of testing each day, the rat was placed directly into the goal box (i.e., escape chamber) for a 4 min adaptation period. After 4 min, the rat was returned to a holding cage, and the goal box was cleaned again with 10% ethanol. The rat was then placed directly in the center of the platform and allowed to explore the maze for 5 min. If the rat found the goal box before time elapsed, the animal was allowed to remain in the chamber for 60 s, and then the trial was stopped, and the rat was returned to its home cage. If the animal did not find the escape chamber, it was simply returned to its home cage at the end of the trial. The maze was surrounded by a series of distinct stationary black and white spatial cues.

### 2.3. Mean Arterial Pressure Measurement and Organ Collection

After behavior tests were completed, the rats underwent surgery, and an indwelling catheter made of V1 and V3 tubing (Scientific Commodities, Lake Havasu City, AZ, USA) was inserted into the carotid artery, tunneled under the skin and exteriorized at the back of the neck, as previously described [27,28]. The rats were allowed to recover and, one day post catheterization, the animals were placed in individual restrainers, and pressure transducers (Cobe III Transducer CDX Sema; Birmingham, AL, USA) were connected to the carotid catheters. Mean arterial pressure was continuously recorded for a 30 min period. Following 48–72 h of mean arterial pressure assessment, the rats were anesthetized, and whole blood was collected, and plasma and serum were saved for future analysis. Maternal organs (brain, kidney, liver and spleen) were collected and stored at −80 °C for further analysis.

### 2.4. Inflammation Assessments

Inflammation was measured in plasma or serum using commercially available enzyme-linked immunosorbent assays. The following proteins were measured: Interleukin-17 (IL-17; R&D Systems), S100B (LifeSpan Biosciences, Seattle, WA, USA) and Brain Derived Neurotrophic Factor (BDNF; Boster Biological, Pleasanton, CA, USA). All assays were run according to the manufacturers’ instructions.

### 2.5. Statistical Analysis

Comparisons between groups (NP, PreE or HELLP), treatment (Orencia vs. no Orencia) or group × treatment interaction were analyzed via one- or two-way analysis of variance (ANOVA) with Tukey’s multiple comparisons tests or Student’s *t*-test with repeated measures when applicable. Data were analyzed with GraphPad Prism 7.02 and expressed as mean ± standard error mean (SEM), where *p* < 0.05 was considered statistically significant.

## 3. Results

### 3.1. Depressive-Like Behavior Is Evident among HDP Dams

**Figure 2:**
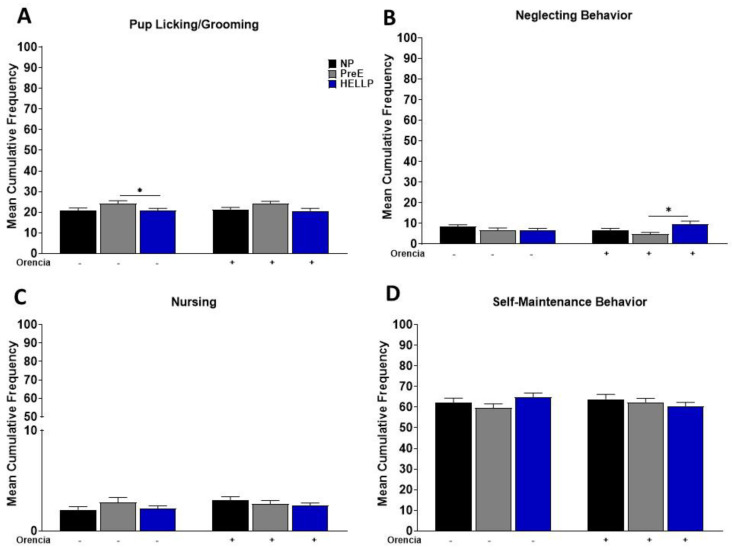
Cumulative frequency of maternal pup interaction from post-partum days 3 to 7 was evaluated. Pup/licking-grooming (**A**), neglecting behavior (**B**), nursing (**C**) and self-maintenance behavior (**D**) were evaluated during both the light and dark period, and data were combined. Data were analyzed by two-way ANOVA followed by Tukey’s post hoc test and presented as mean ± SEM (n = 11–13 dams/group). * denotes *p* < 0.05 in comparison to the indicated group. Abbreviations: NP (normal pregnant); PreE (preeclampsia); HELLP (hemolysis elevated liver enzyme low platelet).

To determine whether HDP alters the maternal care of dams, we observed maternal behavior from post-partum days 3–7. We evaluated the cumulative frequency in which dams spent performing maternal behavior, any contact or care for the pups, as well as non-maternal behavior (self-maintenance). As there were no differences in the cumulative frequency between light cycles or post-partum days (data not shown), the time periods were collapsed, and data reported as mean cumulative frequency per behavior. There was a significant group effect in pup grooming [F_2,255_ = 4.53, *p* = 0.01], with post hoc analysis indicating an increased frequency among PreE relative to HELLP dams (*p* = 0.04, Figure 2A). There was no treatment effect due to Orencia in any of the behaviors; however, there was a group x treatment effect in cumulative frequency [F_2,176_ = 3.51, *p* = 0.03] in neglecting behavior. Post hoc analysis indicated a significant increase in the frequency of this behavior in HELLP + Orencia dams vs. PreE + Orencia dams (*p* = 0.04, Figure 2B). Neither treatment nor group had a significant effect on the cumulative frequency of nursing (*p* = 0.19, *p* = 0.49, Figure 2C) or self-maintenance behaviors (*p* = 0.82, *p* = 0.57, Figure 2D).

**Figure 3:**
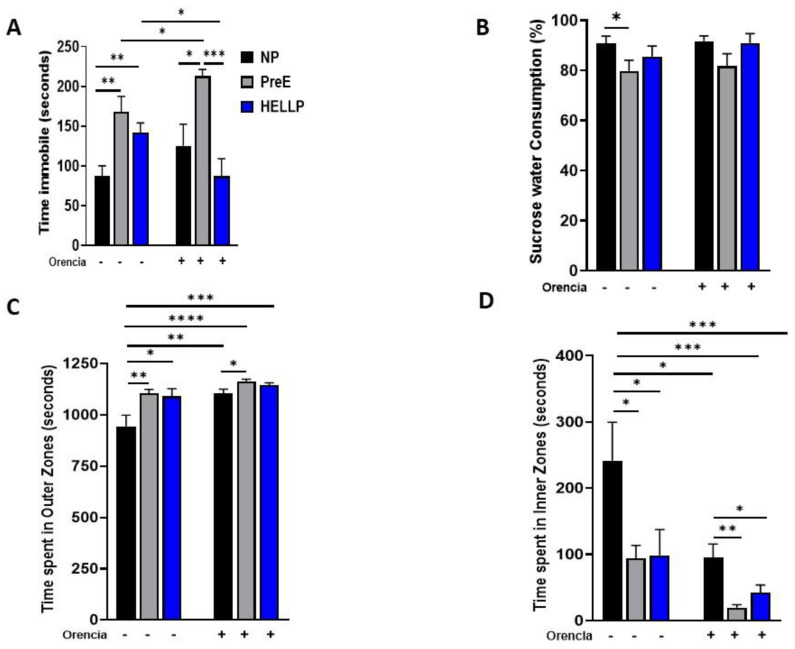
Dams with hypertensive disorders of pregnancy have more immobility and open-area anxiety. Depressive-like behavior, or anhedonia, was assessed in the forced swim test (**A**) and with sucrose preference (**B**). The time spent in the outer zones (**C**) and inner zones (**D**) of the open-field arena was used to assess open-area anxiety. Data were analyzed by two-way ANOVA followed by Tukey’s post hoc test and presented as mean ± SEM (n = 11–13/group). *–**** denotes *p* < 0.05–*p* < 0.00005 in comparison to the indicated group. Abbreviations: NP (normal pregnant); PreE (preeclampsia); HELLP (hemolysis elevated liver enzyme low platelet).

Depressive-like behavior was assessed by measuring immobility in the forced swim test. Immobility time in the forced swim test was evaluated to assess depression. There was a significant group effect [F_2,56_ = 13.56, *p* < 0.0001] and group x treatment effect [F_2,56_ = 4.99, *p* = 0.01] on immobility time for the forced swim test. Both PreE (*p* = 0.002) and HELLP (*p* = 0.006; Figure 3A) dams had increased immobility time relative to NP rats. The infusion of Orencia into HELLP rats significantly reduced the amount of time rats spent in the immobile state compared to uninfused HELLP dams (*p* = 0.04), whereas PreE + Orencia dams spent significantly more time immobile relative to NP + Orencia (*p* = 0.005), HELLP + Orencia (*p* < 0.0001) and PreE rats (*p* = 0.04).

Anhedonia was evaluated via sucrose preference over tap water. There was a significant group effect [F_2,53_ = 3.6, *p* = 0.03] in sucrose preference where PreE rats drank significantly less sucrose relative to NP rats (*p* = 0.04; Figure 3B). There were no significant effects among HELLP rats, regardless of Orencia treatment.

### 3.2. Anxiety-Like Behavior Is Increased in Response to a HDP

The open-field and zero-maze tests were used to evaluate open-space anxiety-like behavior in dams [34]. During the open field, there was a significant group [F_2,68_ = 7.5, *p* = 0.001], treatment [F_1,68_ = 13.22, *p* = 0.0005] and group x treatment [F_2,68_ = 3.19, *p* = 0.05] effect on time spent in the outer zones. NP rats were found to spend significantly less time in the outer zones compared to PreE (*p* = 0.004) and HELLP (*p* = 0.01, Figure 3C) rats and compared to NP + Orencia (*p* = 0.005), PreE + Orencia (*p* < 0.0001) and HELLP + Orencia (*p* = 0.0001) rats. Overall, Orencia infusion increased the time spent in the outer zones; however, only PreE + Orencia rats spent significantly more time in the outer zones relative to NP + Orencia rats (*p* = 0.02). There were similar significant group [F_2,68_ = 7.14, *p* = 0.002], treatment [F_1,68_ = 12.39, *p* = 0.0008] and group x treatment [F_2,68_ = 4.93, *p* = 0.03] effects on time spent in the inner zones of the open field. However, the results were reversed as NP rats spent significantly more time in inner zones of the open field relative to PreE (*p* = 0.02) and HELLP (*p* = 0.03) rats. Orencia-infused rats overall spent less time in the inner zones vs. NP rats (*p* < 0.05; Figure 3D). Among the Orencia rats, both PreE + Orencia (*p* = 0.003) and HELLP + Orencia (*p* = 0.03) rats spent significantly less time in inner zones relative to NP + Orencia rats.

**Figure 4:**
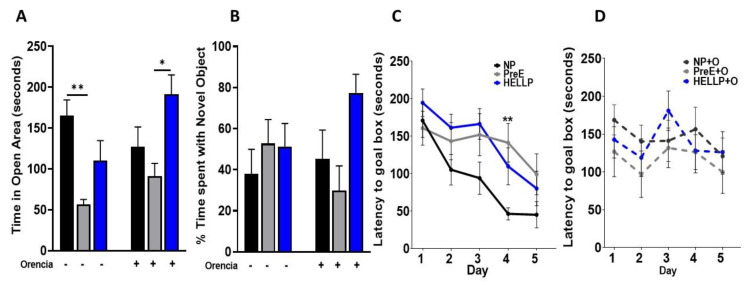
Dams with a hypertensive disorder of pregnancy (HDP) have anxiety and delays in learning. PreE rats spent significantly more time in the open arms of the elevated plus maze, a behavior which was not corrected by Orencia infusion (**A**). There were no significant differences between groups observed in the discrimination index for novel object recognition (**B**). When Barnes maze was assessed over 5 days, only on day 4 were significant differences observed (**C**). However, there was an overall increase in latency to reach the goal box among HDP rats. There was no statistical change in memory due to Orencia administration (**D**). Data were analyzed by two-way ANOVA followed by Tukey’s post hoc test and presented as mean ± SEM (n = 11–13/group). *–** denotes *p* < 0.05–*p* < 0.005 in comparison to the indicated group. Abbreviations: NP (normal pregnant); PreE (preeclampsia); HELLP (hemolysis elevated liver enzyme low platelet).

There was a significant group [F_2,62_ = 9.26, *p* = 0.0003] and group x treatment effect [F_2,62_ = 3.42, *p* = 0.04] on time spent in the open area in the elevated zero maze. Further analysis indicated that PreE rats spent significantly less time in open areas relative to NP rats (*p* = 0.003, Figure 4A). As Orencia lessened the anxiety-like behavior in all groups, PreE + Orencia rats spent significantly less time in the open areas vs. HELLP + Orencia rats (*p* = 0.01, Figure 4A).

### 3.3. Spatial Learning Is Decreased among HDP Rats

Recognition memory, as assessed in the novel object recognition task, was not significantly impacted by hypertension during pregnancy or suppression of T-cell activation by administration of Orencia. There were no significant group [F_2,53_ = 2.71, *p* = 0.08] or treatment effects [F_1,53_ = 0.13, *p* = 0.72] on the discrimination index (Figure 4B).

Spatial learning was assessed in the Barnes maze where there was a significant group [F _2, 368_ = 6.9, *p* = 0.003] and day [F_3.8, 136.8_ = 9.2, *p* < 0.0001] effect in latency to locate the goal box. Latency decreased for all rats over the course of the study; however, on day 4, PreE rats took significantly longer to locate the goal box compared to NP rats (*p* = 0.009, Figure 4C). Orencia treatment did not significantly affect learning and memory among HDP rats (Figure 4D).

### 3.4. HDP Did Not Impact Birth Outcomes but Did Increase Post-Partum Hypertension

There was no significant group [F_2,51_ = 0.48, *p* = 0.62] or treatment [F_1,51_ = 0.12, *p* = 0.73] effect on pup birthweight; neither was there a significant group [F_2,51_ = 0.64, *p* = 0.53] or treatment [F_1,51_ = 0.15, *p* = 0.7] effect on the number of pups born to dams (Table 1). To determine whether hypertension persisted into the post-partum period and whether Orencia treatment during pregnancy had a positive effect on hypertension, mean arterial pressure was assessed after behavioral testing. There was a group [F_2,38_ = 3.3, *p* = 0.04) and group x treatment effect [F_2,38_ = 3.3, *p* = 0.04) on mean arterial pressure. Post hoc analysis indicated that HELLP dams were significantly hypertensive relative to NP (*p* = 0.01) and PreE (*p* = 0.03) dams. Orencia significantly reduced mean arterial pressure in HELLP + Orencia (*p* = 0.04) and PreE + Orencia dams (*p* = 0.002) relative to HELLP dams (Table 1).

### 3.5. S100B Is Increased in PreE Rats, While HDP Decreases BDNF

**Figure 5:**
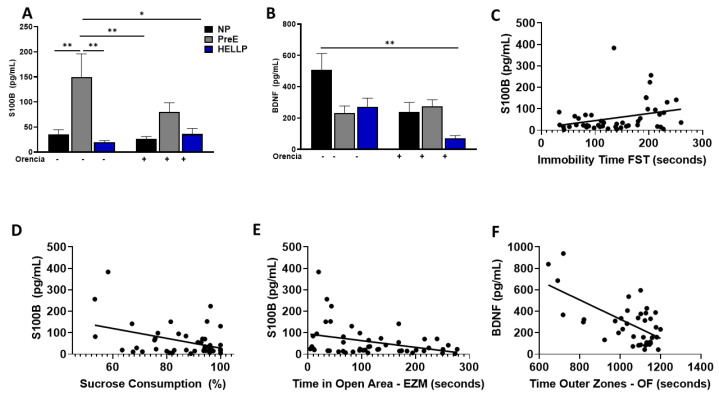
Circulating levels of S100B and BDNF are altered in response to HDP. PreE rats had increased levels of S100B (**A**), and HDP rats had decreased levels of circulating BDNF (**B**) compared to NP rats. Circulating levels of S100B are positively correlated with immobility time in the forced swim test (FST, (**C**)) and negatively correlated with the percent of sucrose consumed (**D**) and time spent in the open arms of the elevated zero maze (EZM, (**E**)), whereas circulating BDNF levels are negatively correlated with the time spent in the outer zones of the open field (OF, (**F**)). On figures (**C**–**F**), the lines represent linear regression, and the circles represent experimental animals. Data were analyzed by two-way ANOVA followed by Tukey’s post hoc test and presented as mean ± SEM (n = 5–8/group), and the correlations were determined via Pearson correlation. *–** denotes *p* < 0.05–*p* < 0.005 in comparison to the indicated group. Abbreviations: NP (normal pregnant); PreE (preeclampsia); HELLP (hemolysis elevated liver enzyme low platelet).

There was a significant group effect [F_2,43_ = 10.94, *p* = 0.0001] on circulating S100B. PreE rats had significantly more levels of S100B vs. NP (*p* = 0.006) and HELLP (*p* = 0.001) rats. Orencia did not have an effect on S100B levels, and PreE rats still had higher levels of S100B relative to both NP + Orencia (*p* = 0.001) and HELLP + Orencia rats (*p* = 0.01, Figure 5A). There were statistically significant group [F_2,32_ = 4.15, *p* = 0.03) and treatment [F_1,32_ = 5.6, *p* = 0.02] effects on circulating BDNF levels. While BDNF levels were decreased in both PreE (*p* = 0.07) and HELLP (*p* = 0.08) rats, statistical significance was not met relative to NP rats. Orencia decreased BNDF levels in all recipients, but only HELLP + Orencia rats had significantly lower levels vs. NP rats (*p* = 0.005, Figure 5B).

To determine whether circulating levels of S100B or BDNF were correlated with anxiety, depression or cognitive ability in the post-partum period, correlation assays were performed on all behavior studies with the exception of maternal pup interaction. S100B had a significant positive correlation with immobility time in the forced swim test (r = 0.294, *p* = 0.04; Figure 5C); however, it was negatively correlated with both total sucrose consumption after 48hrs (r = −0.39, *p* = 0.007, Figure 5D) and with time spent in the open areas of the elevated zero maze (r = −0.345, *p* = 0.02; Figure 5E). There were no other significant correlations between S100B and the remaining behaviors. BDNF levels were negatively correlated with time spent in the outer zones of the open field (r = −0.639, *p* < 0.0001; Figure 5F). There were no significant correlations between BDNF levels and any other behavioral assays.

## 4. Discussion

Hypertension is a risk factor for psychological disorders such as depression and anxiety in non-pregnant populations [35,36,37]. Over the past several years, studies have started to examine the relationship between hypertension during pregnancy, commonly manifested as PreE or HELLP syndrome, and changes in maternal mood and cognition. In the current study, our primary objective was to determine whether rats with a history of HDP had symptoms of depression, anxiety and/or cognitive impairment in the early post-partum period. Our second objective was to determine whether immune suppression during a pregnancy affected by HDP prevented changes in depression, anxiety and/or cognition. Our results indicated that overall HDP negatively influences depression, anxiety and cognition during the early post-partum period and that immune suppression during pregnancy did not offer protection against these negative changes.

HDP had no overall effect on maternal pup interactions. PreE rats had an increase in pup licking/grooming relative to HELLP rats; however, no HDP dams displayed significant differences in any behaviors compared to NP dams. Immobility in the forced swim test has been previously observed in preclinical models used to study post-partum mood disorders [38,39]. HDP dams spent more time immobile in the forced swim test compared to NP rats, suggesting that hypertension during pregnancy contributes to depressive-like behavior or a passive coping response [40]. PreE rats exhibited a decreased preference for sucrose relative to NP rats, whereas HELLP rats had no significant differences in sucrose preference. Collectively, this data indicates that HDP has no effect on maternal care, but it increases depressive-like behavior. This is in agreement with other models of post-partum depression and/or anhedonia, which have demonstrated that animals displaying these characteristics exhibit behaviors akin to that of individuals with post-partum depression [41].

The time spent in the outer zones of the open field correlates with increased anxiety-like behavior. HDP rats were found to spend significantly more time in the outer zones, which conversely led to a significant reduction of time spent in the inner zones of the open field. While complimentary results were also seen in the elevated zero maze, only PreE rats spent significantly less time in the open areas relative to NP rats. To our knowledge, this is the first study to evaluate post-partum anxiety among HDP rats beyond the immediate post-partum period; however, clinical studies do report increases in depression and anxiety among women with HDP [8,42,43]. Furthermore, a study by Ying et al. recently reported that PreE is an independent risk factor for the development of post-partum depression [6]. Thus, our findings support the link between HDP and changes in maternal mood contributing to the development of post-partum depression and/or anxiety.

Some women with a history of a HDP have reported changes in memory and perception [7,9], whereas other studies have not found any differences when verbal learning/memory was evaluated [44,45]. To explore learning and memory in the early post-partum period, we evaluated recognition memory (novel object recognition) and spatial learning and memory (Barnes Maze). There were no differences between groups in respect to recognition memory, and HDP rats showed a trend toward a delay in learning in the Barnes Maze. There are several reasons why we may not have seen changes in cognition, other than the fact that there were no changes due to HDP. More recent studies have reported that only women with PreE plus additional comorbidities have evidence of cognitive impairment in the post-partum period [46], and it has also been suggested that cognitive impairment may appear later in life, coinciding with the time that structural abnormalities are found among women with a history of HDP [45,47]. The results from this current study along with clinical studies indicate the need for further studies evaluating cognition at different time points in the post-partum period.

We also examined circulating neuronal markers associated with anxiety, depression and learning and memory to determine whether there was an association with behaviors. Even though the decrease in BDNF among HDP rats was not significant relative to NP rats, there was a significant negative correlation between BDNF levels and anxiety, which was increased in response to HDP. Similar results were found between S100B levels and anxiety and depressive-like behaviors. These results suggest that there are changes at the neuronal level that are influencing post-partum behavior; however, studies among women with HDP have also found increased levels of S100B and decreased levels of BDNF either during pregnancy or in the immediate post-partum period [48,49,50]. Future work evaluating concentrations of BDNF and S100B in brain regions associated with the behaviors of interest would need to be evaluated to further elucidate the true nature of these relationships.

Our lab and others have previously reported on the relationship between T-cell activation during pregnancy, an increase in mean arterial pressure and subsequent neurovascular damage [27,28,51,52]. Building on our previous work, which reported that a single infusion of Orencia during pregnancy to HELLP rats prevented an increase in blood–brain barrier permeability and decreased anxiety-like behavior in early post-partum rats, we also explored cognition and circulating markers of neuronal injury in the current study [28]. Interestingly, there was no clear benefit to Orencia administration in terms of the behaviors evaluated. Similar to what we previously reported, HELLP + Orencia rats did show positive improvements in anxiety-like behavior, as well as depressive-like behavior in the forced swim test, whereas PreE + Orencia rats consistently performed worse relative to other groups. Overall, these results indicate that a blockade of T cells via Orencia during pregnancy may improve pregnancy outcomes; however, they do not offer any benefits in post-partum mood.

While there were significant differences between PreE and HELLP rats, this is believed to be in part due to the addition of sEng in the HELLP model. sEng, which is believed to work as an anti-angiogenic protein by impairing the binding of transforming growth factor beta-1 to its receptors, is most frequently increased among women with severe PreE and HELLP syndrome. Importantly to the current study, it has been suggested that pharmacological inhibition of transforming growth factor beta-1 inhibits depression [53]. If this is indeed the case, then this could serve as one explanation for why PreE rats performed worse in the forced swim test relative to HELLP rats. Although circulating levels of sFlt-1 and sEng were not evaluated in the current study, several clinical and experimental studies have identified differing results between sFlt-1 and sEng in various cases of HDP [54,55]. However, future larger prospective studies are needed to determine the true role of these anti-angiogenic factors in both the pregnant and post-partum state.

There are several strengths and weaknesses in the current study. Among the strengths are the behavioral assays that were used to assess anxiety, depression and cognition. All of the assays used in the current study are validated behavioral assays and are also used in other rodent models of post-partum depression/anxiety to establish depression or anxiety [38,41,56]. All behavioral testing was overseen by the Institutional animal behavioral core (JPS), which ensured that all behavioral conditions were similar between cohorts and that environmental variables were kept to a minimum. We also used two clinically different models of HDP: PreE and HELLP syndrome. Both PreE and HELLP syndrome are believed to originate from disruptions in the uterine spiral artery formation early in the pregnancy, which leads to downstream placental ischemia [57]. However, despite the similarities in both the origin and maternal comorbidities of these disorders, women affected by PreE and HELLP syndrome have both reported feelings of post-partum depression or anxiety, but at different levels; an effect mirrored in the current study [58]. There have been several clinical studies reporting the onset of post-partum depression/anxiety occurring as early as in the first 3 months among 11.1–19.2% of women and by 1-year post-partum in 6.2–13.1% of women [59,60,61]. With this wide range of time for symptom onset, it is possible that we did not evaluate the correct time frame. However, as the majority of women who do experience post-partum mood changes do so within the immediate post-partum period, we limited our focus to this time frame.

## 5. Conclusions

**Figure 6:**
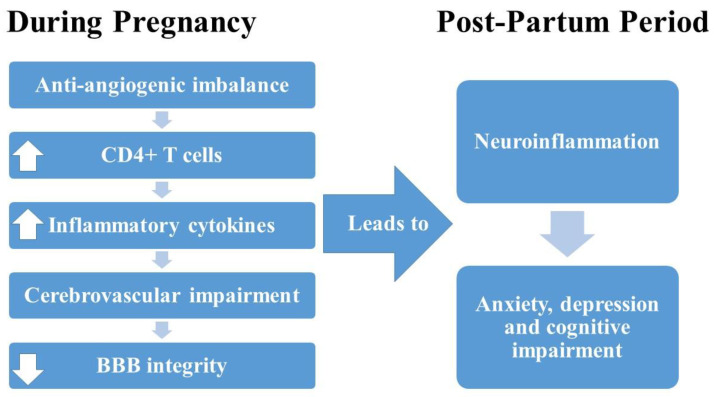
Working hypothesis outlining the relationship between anti-angiogenic imbalance during pregnancy and post-partum changes in mood and cognitive impairment.

This study extends the current knowledge regarding HDP, mood disorders and cognition in preclinical animal models by demonstrating that hypertension and inflammation during pregnancy contributes to long-lasting changes in maternal mood and cognition. Additionally, the distinct psychological and immunological differences between the PreE (sFlt-1 infusion) and HELLP (sFlt-1 and sEng infusion) models suggest different mechanisms of action within the different models, similar to what is seen clinically. We also evaluated the utility of immune suppression via T-cell blockade on improving maternal mood and cognition in our preclinical animal models. Unfortunately, it does not appear as if the regimen of T-cell blockade used in the current study has a role in neuroprotection. These studies do emphasize the need for future studies to evaluate the safety and effectiveness of pharmacotherapy for mental health both during pregnancy and in the post-partum period. As outlined in our current working hypothesis (Figure 6), anti-angiogenic imbalance leads to increased CD4^+^ T cells, leading to increased production of inflammatory cytokines, cerebrovascular impairment and decreased integrity of the blood–brain barrier, all of which leads to neuroinflammation, which contributes to the development of anxiety, depression and cognitive impairment.

In summary, the connection between HDP and mood disorders is still somewhat unclear; however, inflammation has been proposed to play a large role. Conversely, the connection between HDP and inflammation and brain damage are somewhat more defined. The combination of this study and clinical studies point to significant changes in maternal mood following HDP, providing more evidence for the need for increased mental health screening during pregnancy and the post-partum period. This is especially true for women who are at risk of HDP or who have already been diagnosed with one.

## Figures and Tables

**Table 1:** Birth outcomes and mean arterial pressure for dams in the current study. Birth outcomes were collected on post-partum day 0, which was marked by the delivery of rat pups. Mean arterial pressure was collected at the end of the study at post-partum week 9.

Variables	NP	PreE	HELLP	NP + O	PreE + O	HELLP + O	*p* Value ^1^
Pup Birth Wt (g)	7.05 ± 0.03	7.1 ± 0.2	6.7 ± 0.2.	6.7 ± 0.2	7.1 ± 0.2	7.2 ± 0.2	0.62
Litter #	9.7 ± 1	9.8 ± 0.8	9.5 ± 0.9	10.8 ± 0.7	8.7 ± 0.8	10.3 ± 1.3	0.53
MAP (mmHg)	111.3 ± 3.9 ^a^	116 ± 3.6 ^a^	127.1 ± 2.6	118.3 ± 3.5	111.75 ± 3.2 ^a^	117 ± 3.7	0.01

^a^ denotes significant relationship compared to HELLP; ^1^ *p* value is representative of group effect from two-way ANOVA. Abbreviations: NP (normal pregnant); PreE (preeclampsia); HELLP (hemolysis elevated liver enzyme low platelet); O (Orencia); MAP (mean arterial pressure).

## Data Availability

Data are contained within the article.

## References

[1] Hutcheon J., Lisonkova S., Joseph K. (2011). Epidemiology of pre-eclampsia and the other hypertensive disorders of pregnancy. Best Pract. Res. Clin. Obstet. Gynaecol..

[2] Febres-Cordero D., Young B. (2021). Hypertensive Disorders of Pregnancy. Neoreviews.

[3] Engelhard I., Van Rij M., Boullart I., Ekhart T., Spaanderman M., Van Den Hout M., Peeters L. (2002). Posttraumatic stress disorder after pre-eclampsia: An exploratory study. Gen. Hosp. Psychiatry.

[4] Baecke M., Spaanderman M., Van Der Werf S. (2009). Cognitive function after pre-eclampsia: An explorative study. J. Psychosom. Obstet. Gynaecol..

[5] Stramrood C., Wessel I., Doornbos B., Aarnoudse J., Van Den Berg P., Schultz W., Van Pampus M. (2011). Posttraumatic stress disorder following preeclampsia and PPROM: A prospective study with 15 months follow-up. Reprod. Sci..

[6] Ye Y., Chen L., Xu J., Dai Q., Luo X., Shan N., Qi H. (2021). Preeclampsia and its complications exacerabate development of postpartum depression: A Retrospective Cohort Study. BioMed Res. Int..

[7] Brusse I., Duvekot J., Jongerling J., Steegers E., De Koning I. (2008). Impaired maternal cognitive functioning after pregnancies complicated by severe pre-eclampsia: A pilot case-control study. Acta Obstet. Gynecol. Scand..

[8] Chapuis-de-Andrade S., Moret-Tatay C., De Paula T., Irigaray T., Antonello I., da Costa B. (2022). Psychological factors and coping strategies in pregnancies complicated by hypertension: A cluster-analytic approach. J. Affect. Disord..

[9] Fields J., Garovic V., Mielke M., Kantarci K., Jayachandran M., White W., Butts A., Graff-Radford J., Lahr B., Bailey K. (2017). Preeclampsia and cognitive impairment later in life. Am. J. Obstet. Gynecol..

[10] Miller K., Miller V., Barnes J. (2019). Pregnancy History, Hypertension, and Cognitive Impairment in Postmenopausal Women. Curr. Hypertens. Rep..

[11] Habli M., Eftekhari N., Wiebracht E., Bombrys A., Khabbaz M., How H., Sibai B. (2009). Long-term maternal and subsequent pregnancy outcomes 5 years after hemolysis, elevated liver enzymes, and low platelets (HELLP) syndrome. Am. J. Obstet. Gynecol..

[12] Mommersteeg P., Drost J., Ottervanger J., Maas A. (2016). Long-term follow-up of psychosocial distress after early onset preeclampsia: The Preeclampsia Risk Evaluation in FEMales cohort study. J. Psychosom. Obstet. Gynaecol..

[13] Hoedjes M., Berks D., Vogel I., Franx A., Bangma M., Darlington A., Visser W., Duvekot J., Habbema J., Steegers E. (2011). Postpartum depression after mild and severe preeclampsia. J. Womens Health.

[14] Blom E., Jansen P.V., Verhulst F.C., Hofman A., Raat H., Jaddoe V., Coolman M., Steegers E., Tiemeier H. (2010). Perinatal complications increase the risk of postpartum depression. The Generation R Study. Br. J. Obstet. Gynaecol..

[15] Adank M., Hussainalli R., Oosterveer L., Ikram M., Steegers E., Miller E., Schalekamp-Timmermans S. (2021). Hypertensive Disorders of Pregnancy and Cognitive Impairment: A Prospective Cohort Study. Neurology.

[16] Shaaban C., Rosano C., Cohen A., Huppert T., Butters M., Hengenius J., Parks W., Catov J. (2021). Cognition and cerebrovascular reactivity in midlife women with history of preeclampsia and placental evidence of maternal vascular malperfusion. Front. Aging Neurosci..

[17] Amaral L., Wallace K., Owens M., Lamarca B. (2017). Pathophysiology and Current Clinical Management of Preeclampsia. Curr. Hypertens. Rep..

[18] Wallace K., Harris S., Addison A., Bean C. (2018). HELLP Syndrome: Pathophysiology and Current Therapies. Curr. Pharm. Biotechnol..

[19] Margioula-Siarkou G., Margioula-Siarkou C., Petousis S., Margaritis K., Vavoulidis E., Gullo G., Alexandratou M., Dinas K., Sotiriadis A., Mavromatidis G. (2021). The role of endoglin and its soluble form in pathogenesis of preeclampsia. Mol. Cell. Biochem..

[20] Maynard S., Karumanchi S. (2011). Angiogenic factors and preeclampsia. Semin. Nephrol..

[21] Wallace K., Morris R., Kyle P., Cornelius D., Darby M., Scott J., Moseley J., Chatman K., Lamarca B. (2014). Hypertension, inflammation and T lymphocytes are increased in a rat model of HELLP syndrome. Hypertens. Pregnancy.

[22] Wallace K., Martin J.N., Tam K.T., Wallukat G., Dechend R., Lamarca B., Owens M. (2013). Seeking the Mechanisms of Action for Corticosteroids in HELLP Syndrome: SMASH Study. Am. J. Obstet. Gynecol..

[23] Rana S., Burke S., Karumanchi S. (2020). Imbalances in circulating angiogenic factors in the pathophysiology of preeclampsia and related disorders. Am. J. Obstet. Gynecol..

[24] Amburgey O., Chapman A., May V., Bernstein I., Cipolla M. (2010). Plasma from preeclamptic women increases blood-brain barrier permeability. Hypertension.

[25] Wallace K., Tremble S., Owens M., Morris R., Cipolla M. (2015). Plasma from patients with HELLP Syndrome Increases Blood-brain barrier permeability. Reprod. Sci..

[26] Euser A., Bullinger L., Cipolla M. (2008). Magnesium sulphate treatment decreases blood-brain barrier permeability during acute hypertension in pregnant rats. Exp. Physiol..

[27] Bean C., Spencer S., Bowles T., Kyle P., Williams J., Gibbens J., Wallace K. (2016). Inhibition of T cell-activation attenuates hypertension, TNF-alpha, IL-17 and blood-brain barrier permeability in pregnant rats with angiogenic imbalance. Am. J. Reprod. Immunol..

[28] Wallace K., Bean C., Bowles T., Spencer S., Randle W., Kyle P., Shaffery J. (2018). Hypertension, Anxiety, and Blood-Brain Barrier Permeability are Increased in post-partum rats with a history of Severe Preeclampsia/Hemolysis, Elevated Liver Enzymes and Low Platelet Syndrome. Hypertension.

[29] Novotny S., Wallace K., Herse F., Moseley J., Darby M., Heath J., Gill J., Wallukat G., Martin J., Dechend R. (2013). CD4+ T cells play a critical role in mediating hypertension in response to placental ischemia. J. Hypertens..

[30] Chourbaji S., Hoyer C., Richter S., Brandwein C., Pfeiffer N., Vogt M., Vollmayr B., Gass P. (2011). Differences in mouse maternal care behavior—Is there a genetic impact of the glucocorticoid receptor?. PLoS ONE.

[31] Castagne V., Moser P., Roux S., Porsolt R. (2011). Rodent models of depression: Forced swim and tail suspension behavioral despair tests in rats and mice. Curr. Protoc. Neurosci..

[32] Sutcliffe J., Marshall K., Neill J. (2007). Influence of gender on working and spatial memory in the novel object recogntion task in the rat. Behav. Brain Res..

[33] Barnes C. (1979). Memory deficits associated with senescence: A Neurophysiological and behavioral study in the rat. J. Comp. Physiol. Psychol..

[34] Salari A., Fatehi-Gharehlar L., Motayagheni N., Homberg J. (2016). Fluoxetine normalizes the effects of prenatal maternal stress on depression- and anxiety-like behaviors in mouse dams and male offspring. Behav. Brain Res..

[35] Kessler R., Ormel J., Demler O., Stang P. (2003). Comorbid mental disorders account for the role impairment of commonly occurring chronic physical disorders: Results from the National Comorbidity Survey. J. Occup. Environ. Med..

[36] Bussotti M., Sommaruga M. (2018). Anxiety and depression in patients with pulmonary hypertension: Impact and management challenges. Vasc. Health Risk Manag..

[37] McConnell S., Jacka F., Williams L., Dodd S., Berk M. (2005). The relationship between depression and cardiovascular disease. Int. J. Psychiatry Clin. Pract..

[38] Li M., Chou S.-Y. (2016). Modeling postpartum depression in rats: Theoretic and methodological issues. Dongwuxue Yanjiu.

[39] Perani C., Slattery D. (2014). Using animal models to study post-partum psychiatric disorders. Br. J. Pharm..

[40] Molendijk M., De Kloet E. (2019). Coping with the forced swim stressor: Current state-of-the-art. Behav. Brain Res..

[41] Navarre B., Laggart J., Craft R. (2010). Anhedonia in postpartum rats. Physiol. Behav..

[42] Araji S., Griffin A., Dixon L., Spencer S., Peavie C., Wallace K. (2020). An Overview of Maternal Anxiety During Pregnancy and the Post-partum Period. J. Ment. Health Clin. Psychol..

[43] Van Esch J., Bolte A., Vandenbussche F., Schippers D., De Weerth C., Beijers R. (2020). Differences in hair cortisol concentrations and reported anxiety in women with preeclampsia versus uncomplicated pregnancies. Pregnancy Hypertens..

[44] Papousek I., Weiss E., Moertl M., Schmid-Zaludek K., Krenn E., Lessiak V., Lackner H. (2021). Unaffected memory and inhibitory functioning several weeks postpartum in women with pregnancy complicated by preeclamspia. Behav. Sci..

[45] Elharram M., Dayan N., Kaur A., Landry T., Pilote L. (2018). Long-term cognitive impairment after preeclampsia: A systematic review and meta-analysis. Obstet. Gynecol..

[46] Bergman L., Thorgeirsdottir L., Elden H., Hesselman S., Schell S., Ahlm E., Aukes A., Cluver C. (2021). Cognitive impairment in preeclampsia complicated by eclampsia and pulmonary edema after delivery. Acta Obstet. Gynecol. Scand..

[47] Ijomone O., Shallie P., Naicker T. (2018). Changes in the structure and function of the brain years after pre-eclampsia. Ageing Res. Rev..

[48] Wu J., Sheng X., Zhou S., Fang C., Song Y., Wang H., Jia Z., Jia X., You Y. (2021). Clinical significance of S100B protein in pregnant woman with early- onset severe preeclampsia. Ginekol. Pol..

[49] Artunc-Ulkumen B., Guvnec Y., Goker A., Gozukara C. (2015). Maternal serum S100-B, PAPP-A and IL-6 levels in severe preeclampsia. Arch. Gynecol. Obstet..

[50] Perucci L., Vieira E., Teixeira A., Gomes K., Dusse L., Sousa L. (2017). Decreased plasma concentrations of brain-derived neurotrophic factor in preeclampsia. Clin. Chim. Acta.

[51] Wallace K., Cornelius D., Scott J., Heath J., Moseley J., Chatman K., LaMarca B. (2014). CD4^+^ T cells are important mediators of oxidative stress that cause hypertension in response to placental ischemia. Hypertension.

[52] Bean C., Spencer S., Pabbidi M., Szczepanski J., Araji S., Dixon S., Wallace K. (2018). Peripheral anti-angiogenic imbalance during pregnancy impairs myogenic tone and increases cerebral edema in a rodent model of HELLP Syndrome. Brain Sci..

[53] Hong M., Zheng J., Ding Z., Chen J., Yu L., Niu Y., Hua Y., Wang L. (2013). Imbalance between Th17 and Treg cells may play an important role in the development of chronic unpredictable mild stress-induced depression in mice. Neuroimmunomodulation.

[54] Parrish M., Murphy S., Rutland S., Wallace K., Wenzel K., Wallukat G., Keiser S., Ray L., Dechend R., Martin J. (2010). The effect of immune factors, Tumor Necrosis Factor-alpha, and agonistic autoantibodies to the Angiotensin II Type I Receptor on Soluble fms-Like Tyrosine-1 and Soluble Endoglin production in response to hypertension during pregnancy. Am. J. Hypertens..

[55] Venkatesha S., Toporsian M., Lam C., Hanai J., Mammoto T., Kim Y., Bdolah Y., Lim K., Yuan K., Libermann T. (2006). Soluble endoglin contributes to the pathogenesis of preeclampsia. Nat. Med..

[56] Yang Y., Zhao S., Yang X., Lie W., Si J., Yang X. (2022). The antidepressant potential of lactobacillus casei in the postpartum depression rat model mediated by the microbiota-gut-brain axis. Neurosci. Lett..

[57] Campos A., Goncalves A., Massa A., Amaral P., Silva P., Aguilar S. (2016). HELLP Syndrome a severe form of preeclampsia: A comparative study of clinical and laboratorial parameters. Am. J. Exp. Clin. Res..

[58] Roberts L., Davis G., Horner C. (2019). Depression, anxiety and post-traumatic stress disorder following a hypertensive disorder of pregnancy: A narrative literature review. Front. Cardiovasc. Med..

[59] Gavin N., Gaynes B., Lohr K., Meltzer-Brody S., Gartlehner G., Swinson T. (2005). Perinatal depression: A systematic review of prevalence and incidence. Obstet. Gynecol..

[60] Rubertsson C., Wickberg B., Gustavsson P., Radestad I. (2005). Depressive symptoms in early pregnancy, two months and one year postpartum—Prevlance and psychosocial risk factors in a national Swedish sample. Arch. Womens Ment. Health.

[61] Kikuchi S., Murakami K., Obara T., Ishikuro M., Ueno F., Noda A., Onuma T., Kobayashi N., Sugawara J., Yamamoto M. (2021). One-year trajectories of postpartum depressive symptoms and associated psychosocial factors: Findings from the Tohoku Medical Megabank Project Birth and Three-Generation Cohort Study. J. Affect. Disord..

